# Mental Health Intent Recognition for Arabic-Speaking Patients Using the Mini International Neuropsychiatric Interview (MINI) and BERT Model

**DOI:** 10.3390/s22030846

**Published:** 2022-01-23

**Authors:** Ridha Mezzi, Aymen Yahyaoui, Mohamed Wassim Krir, Wadii Boulila, Anis Koubaa

**Affiliations:** 1 Military Academy of Fondouk Jedid, Nabeul 8012, Tunisia; aymen.yahyaoui@ept.rnu.tn; 2 SERCOM Laboratory, University of Carthage, Carthage 1054, Tunisia; 3 Department of Psychiatry, Military Hospital of Instruction of Tunis, Mont Fleury, Tunis 1008, Tunisia; wassim.krir@outlook.com; 4 Robotics and Internet-of-Things Laboratory, Prince Sultan University, Riyadh 12435, Saudi Arabia; wadii.boulila@riadi.rnu.tn (W.B.); akoubaa@psu.edu.sa (A.K.); 5 RIADI Laboratory, National School of Computer Science, University of Manouba, Manouba 2010, Tunisia

**Keywords:** mental health, psychiatry, MINI, intent recognition, BERT model, natural language processing, machine learning

## Abstract

For many years, mental health has been hidden behind a veil of shame and prejudice. In 2017, studies claimed that 10.7% of the global population suffered from mental health disorders. Recently, people started seeking relaxing treatment through technology, which enhanced and expanded mental health care, especially during the COVID-19 pandemic, where the use of mental health forums, websites, and applications has increased by 95%. However, these solutions still have many limits, as existing mental health technologies are not meant for everyone. In this work, an up-to-date literature review on state-of-the-art of mental health and healthcare solutions is provided. Then, we focus on Arab-speaking patients and propose an intelligent tool for mental health intent recognition. The proposed system uses the concepts of intent recognition to make mental health diagnoses based on a bidirectional encoder representations from transformers (BERT) model and the International Neuropsychiatric Interview (MINI). Experiments are conducted using a dataset collected at the Military Hospital of Tunis in Tunisia. Results show excellent performance of the proposed system (the accuracy is over 92%, the precision, recall, and F1 scores are over 94%) in mental health patient diagnosis for five aspects (depression, suicidality, panic disorder, social phobia, and adjustment disorder). In addition, the tool was tested and evaluated by medical staff at the Military Hospital of Tunis, who found it very interesting to help decision-making and prioritizing patient appointment scheduling, especially with a high number of treated patients every day.

## 1. Introduction

It is no secret that mental health and physical health are handled inequitably, leading to many obstacles. This discrepancy can take multiple forms, from divisive cultural attitudes to mental health care coverage discrimination. Thus, imbalances in outcomes emerge from the disparity between psychiatric care and physical disorders. However, technology has opened up a new field in mental health support and data gathering in recent years, which could be a safer first option for those who have previously stopped seeking mental health treatment. Some technologies may be more attractive than conventional therapy approaches, and they may provide mental health patients with advanced treatment options [1]. Thousands of published papers per year, hundreds of different applications, tools, and frameworks tackle the challenges and open issues related to mental health. The MINI-International Neuropsychiatric Interview (MINI) [2] and the machine learning (ML) algorithms for natural language processing (NLP) [3] are among these tools. MINI is an easy, multilingual, and well-organized tool to diagnose common mental health issues. Using its Tunisian Arabic version in a mental health application is a good option as it has proven its proficiency for many years. The ML model, bidirectional encoder representations from transformers (BERT) [4], is also a great NLP tool [5,6].

In this work, we propose a mental health diagnosis application for Arabic-speaking patients using both MINI and the supervised machine learning BERT model to equip the psychiatry department of the Military Hospital of Instruction of Tunis with a rapid and intelligent tool handling the high number of patients treated every day. This tool permits to assess treatment priority and appointment dates based on patients’ states severity.

The main contributions of this paper are summarized as follows:

- It presents an overview of the different state-of-the-art techniques, methods, and tools related to AI mental health diagnosis.
- It treats the specific use case of Arabic-speaking patients using a combination of MINI and an adapted BERT model for mental health intent recognition by taking the written or spoken input of a patient and classifying it according to a defined diagnosis, which leads to accurate results with the use of a very unique and first-of-its-kind dataset, built from scratch using the Tunisian dialect phrases with Arabic letters.
- It presents our interactive tool developed for the specific needs of the medical staff at the military hospital of Tunisia psychiatry department. This tool was deployed there and tested on many patients. The medical staff confirmed it as an interesting and valuable tool for patient state assessment and filtering and more efficient appointment management. To our knowledge, it is the first of its kind of mental health application in the country.

The rest of this paper is organized as follows. In Section 2, we present the dilemma of mental health issues in modern days and their impact on people’s wellbeing alongside a brief explanation of MINI, its use, and its different scenarios that we dealt with in our work. In addition, this section presents general concepts about artificial intelligence (AI) in mental health diagnosis and NLP and BERT models. Section 3 provides a detailed review of the current state of the art of a set of highly relevant research works and tools that tackle the AI and machine learning use in healthcare and mental health.In addition, it reviews the use of NLP, especially with the BERT model, in making a mental health diagnosis. Section 4 presents our mental health proposed diagnosis system for Arabic-speaking patients (Tunisian dialect) based on MINI and BERT model for NLP to ensure an accurate diagnosis. Section 5 is dedicated to evaluating our proposed solution and analyzing its proficiency, performance, and accuracy results. Section 6 is a discussion of the system’s main strengths and limits. Finally, Section 7 concludes the paper and presents its perspectives.

## 2. Background

This section introduces the mental health issues and presents MINI and general concepts about AI in mental health diagnosis and NLP and BERT models.

### 2.1. Mental Health Issues

At some point, every person feels disturbed, anxious, or even downhearted, leading to significant mental health issues. According to the ICD-11 [7] as a reference for mental health illnesses, the identification of a mental health illness is often related to mental problems disturbing thinking ability, relation with others, and day-to-day behaviors.

Several mental issues have been studied. Most of these studies focused mainly on schizophrenia, stress disorder, depression, bipolar disorder, obsessive-compulsive disorder, and others [8].

Accordingly, mental issues are global problems and equal opportunity issues. They equally affect the young and the old, male or female, every race, ethnic group, and different education and income levels.

There has been considerable talk about how new settings generate a depression pandemic [9]. More than 200 million people of different age categories are suffering depression according to WHO (World Health Organization) [10]. In addition, up to 20% of children and teenagers worldwide suffer from mental health issues. Moreover, they are believed to impact one out of every four people at some point in their lives. As a result, issues such as alcohol and substance addiction, abuse, and gender-based violence emerge and influence mental health. Thus, failing to treat mental health has ramifications for entire civilizations. These disturbing statistics indicate the widespread incidence of mental illness. The good news is that they are frequently treatable.

Although symptoms of mental illness might manifest themselves physically in the form of stomach pain and back pain in some people, other symptoms do not include physical pain; among these symptoms are the following:

- Feeling down for awhile.
- Severe mood fluctuations.
- Avoiding contact with family and friends.
- Decreasing energy or difficulty sleeping.
- Feeling enraged, aggressive, or violent regularly.
- Having hallucinations, hearing unreal voices, or feeling paranoid.
- Having thoughts about ending their lives or death.

Hence, consulting a therapist and sticking to a regular treatment plan that may involve medication can help people with mental illnesses feel better and reduce their symptoms.

### 2.2. MINI International Neuropsychiatric Interview

Performing a psychiatric interview necessitates a set of questions to ask the patient. “MINI PLUS” (MINI International Neuropsychiatric Interview) [2] is a commonly used psychiatric structured diagnostic interview instrument that requires “yes” or “no” responses, and it is divided into modules where each module has a set of questions to ask the patient. The questions are labeled with letters that relate to diagnostic groups. For instance, module 1 checks if the patient has a depressive episode using questions A1a, A1b, A2a, A2b, etc. (e.g., question A1a: Have you been consistently depressed or down, most of the day, nearly every day, over the past two weeks?). According to the patient’s answers, there is a specific diagnosis at the end of each module. It is considered a gold standard for AI because it is very structured, and all its questions are known. We can easily predict all of their answers even if they are other than “yes” and “no”. Accordingly, it made it easy to build our dataset to test and train the machine learning models. The interview works on many modules such as depression, suicidality, panic disorders, social phobia, etc. However, in our case, trying to make the interview shorter than a real one, we tested the five most frequent and common disorders or modules according to the WHO [11], which are depression, suicidality, panic disorder, social phobia, and adjustment disorder and tried to implement them in our application.

#### Different Scenarios While Taking the Test with MINI

**Figure 1:**
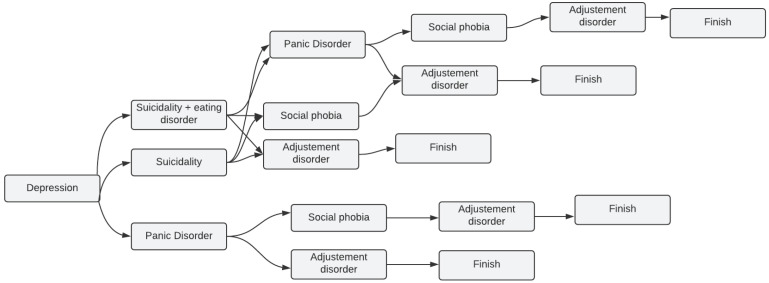
Most common scenarios of depression using MINI in our system.

In our application, we did not take the MINI by letter. Instead, we took the modules we needed to work with and tried to implement them as we could see fit with the majority of cases we are dealing with, which led to some common scenarios with only a few disorders among the five mentioned in Section 2.2. After all, it is rare that a person has all the diseases at once. Figure 1 depicts all the possible scenarios a patient might go through while taking the application test from start to finish.

The following is an example of how the scenario might unfold when using the MINI modules in our system:

- According to the patient’s answers, while taking the depression test, it turned out that the patient needed suicidality and eating disorder test, the trial went to the next module, which is, in our case, suicidality and eating disorder.
- In some other cases, if the patient from the beginning of the test is not identified as having the issue related to the tested module (one of the five modules), the test jumps to start the next one. For instance, if the patient from the beginning does not have panic disorder by answering the first two questions of the module with “No” or “Not”, according to the MINI, the test jumps to the social phobia test.

### 2.3. AI and ML in Healthcare and Mental Health

While many different fields of society are willing to embrace the potential of AI, caution remains deep in medical areas, among which are psychiatry and mental health, proven by recent headlines in the news such as “Warnings of a Dark Side to AI in Health Care” [12]. Psychiatry is a promising area for the use of AI, though, despite the claimed worries, AI implementations in the medical field are progressively expanding. Thus, we are compelled to apprehend its present and future applications in mental health and work intelligently with AI as it enters the clinical mainstream.

### 2.4. NLP with BERT

NLP is the automated manipulation of natural language by software, such as speech and text. It has been studied for more than 50 years, and it sprang from the discipline of linguistics as computers became more prevalent. Most works use convolutional neural networks (CNN) and recurrent neural networks (RNN) to achieve NLP functions [13]. A novel architecture [14] has advanced existing classification tasks by using deep layers that are commonly used in computer vision to perform text processing. They concluded that adding more depth to the model would improve its accuracy. It was the first time deep convolutional networks have been used in NLP, and it has provided insight into how it can help with other activities. Opinion mining, also known as sentiment analysis, is another used field. It is a primary method for analyzing results. For text preprocessing, NLP strategies are checked, and opinion mining methods are studied for various scenarios [15]. Human language can be learned, understood, and generated using NLP techniques. Speaking conversation networks and social media mining are examples of real-world applications [16]. As the purpose of this paper is a mental health diagnosis system for Arabic-speaking patients, we will deal with Arabic text (in Tunisian dialect “Darija”) from right to left, which makes this model the perfect choice to achieve the ultimate results. After all, we are dealing with a medical condition where high-quality results cannot be less important.

**Figure 2:**
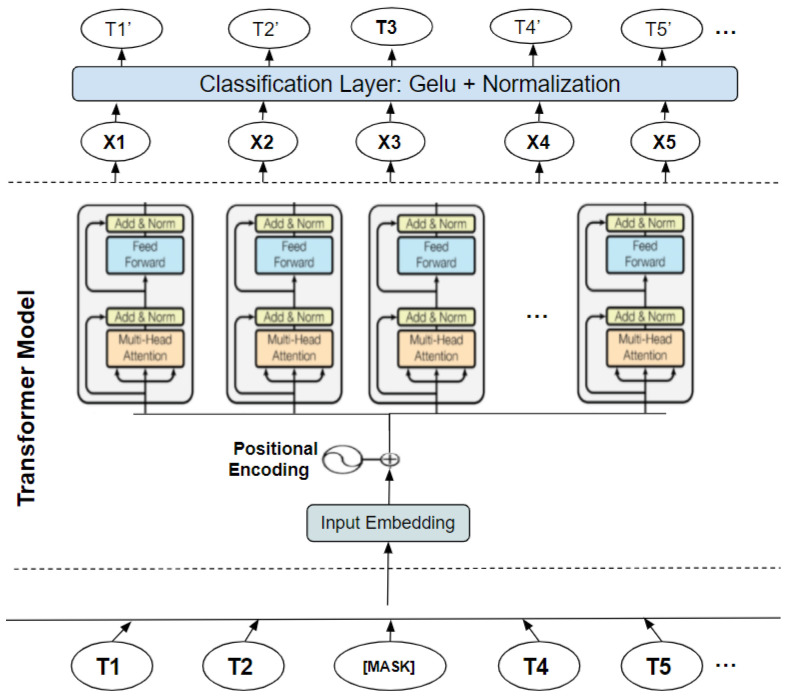
BERT architecture.

BERT is a multilingual transformer-based ML technique for NLP pre-training developed by Google [4]. It has sparked debate in the ML field by showing cutting-edge findings in a wide range of NLP tasks, such as question answering, natural language inference, and others. The transformer’s bidirectional training to language modeling is the cornerstone of BERT’s technological breakthrough, which contains two distinct mechanisms: an encoder that reads text input and a decoder that generates a task prediction. Only the encoder technique is required because BERT’s objective is to create a language model. In contrast, previous research has focused on text sequences from the left to the right or the left (directional models). However, the transformer encoder scans the complete word sequence in one go. Accordingly, it is considered bidirectional, although it is more appropriate to be described as nondirectional. This feature enables the model to divine the context of a word from its surrounds (on the left and the right of the word) and gain a superior understanding of language context and flow more than single-direction language models. The transformer encoder is described in detail in Figure 2. A series of tokens integrated into vectors are the input to be processed by the neural network. The final result is an H-dimensional series of vectors. Each of these vectors corresponds to the same index input word [17].

## 3. Related Works

Over the last decades, various works and developed applications tackling real-life AI usages have emerged in the scientific world. These applications are related to psychotherapy as chatterbots, virtual reality-based systems, and clinician systems.

### 3.1. ChatterBots

Chatterbots are computer tools developed to interact with one or multiple users in simulated intelligent conversations [18]. The most well-known ones in mental health diagnosis and therapy are Eliza, Parry, Youper, Woebot, Moodkits, and Wysa.

#### 3.1.1. The ChatterBot ELIZA

The ChatterBot ELIZA is the primary chatterbot combining AI and psychotherapy [19]. It was developed in the 1960s as an NLP program made to reenact discussions with users and provide them with the feeling and the illusion that they were talking to someone who understood them. It was an exceptionally imperative and fruitful test, which led to various other bots. Nevertheless, the main goal of this software is to imitate a psychologist having an interaction with a patient. However, it could not perform recommendations and provide solutions to patients’ problems.

#### 3.1.2. The ChatterBot PARRY

Psychiatrist Colby [20] considered that a computer is a better way of studying the disease than an actual patient. Thus, he created a system at Stanford University named PARRY in the early 1970s, a bot that tried to model the behavior of a paranoid schizophrenic and could converse with others, such as ELIZA. PARRY was praised for being the first Turing test program for judging computer intelligence after Alan Turing [21]. A computer machine imitates a human writing dialogue with a human judge in real time.

#### 3.1.3. Youper

Youper [22] is a chatbot software developed in 2016 as an AI tool to help users recognize, monitor, and process their emotions and thoughts. Cognitive behavioral therapy (CBT), recognition and engagement therapy (ACT), and mindfulness can enhance mood and sleep habits and alleviate anxiety, stress, and depression symptoms. Youper the AI chatbot, journal logs, attitude logs, and a mental wellbeing appraisal are the four key features of the software. Users may ask the Youper chatbot to help them define their feelings by explaining how they are feeling right now. The consumer is then asked to describe the causes of incidents that have led to their present state of mind. Finally, Youper recommends a few things to help make the most of the remainder of the day. The journal log gathers all users’ answers to the Youper chatbot’s “How are you feeling?” question. The mood log feature captures user answers to the Youper chat “What makes you feel this way?” prompts.

#### 3.1.4. Woebot

Woebot is a blend of AI, chatbot, and cognitive behavioral therapy (CBT) developed by Stanford researchers. The AI application assists users in self-managing their mental health difficulties [23]. The commercial edition of the application includes unique features such as a subscription model for conversations, mood tracking, word games, and engaging with users who desperately need assistance.

#### 3.1.5. Moodkit

MoodKit is a smartphone application meant for managing depression, anxiety, and stress and uses the CBT methods. It is applied for checking thought, tracking the mood, and the schedule of activities, on a set of instructions while using text-based information to interact with the person [24].

#### 3.1.6. Wysa

Wysa [25] is an AI-based, emotionally intelligent chatbot. It helps manage thoughts and emotions via a combination of tools and techniques such as dialectical behavior therapy (DBT), evidence-based CBT, and guided meditation.

### 3.2. Virtual Reality Works

#### 3.2.1. Virtual Reality Human Avatars

Virtual reality human avatar-based AI is likely used in mental health services with all such forms of person-to-person experiences, including psychiatric therapies, tests, and monitoring. The use of virtual reality avatars to provide knowledge and support to people about mental health services is now in use [26]. For example, SimCoach is intended to link members of the armed service and their broods to mental health care and other support tools [26].

One day, this form of AI technology can revolutionize telepractice; AI-enabled avatars can be remotely accessible and deliver therapeutic services. Among its advantages is the ability to easily access the digital AI-enabled interactive consultations by patients. It can offer simple examinations, advice, and referrals for additional care. An essential advantage of using avatar programs for virtual reality is ensuring people’s privacy by providing them treatment using a virtual manner.

The AI-based avatar applications are more dynamic and entertaining, which provides more easiness in their use. These programs often can assist clinicians by serving professional experts who have the expertise of specialized areas or subjects, as always possible, similar to how we intend to do in this project.

#### 3.2.2. Applications Based on Virtual Reality in Mental Healthcare

Nowadays, there is an increasing interest in virtual reality in healthcare. This form of treatment enables patients to simulate a natural environment in which they can dive and explore a new world [27]. This technology can be of great importance for assessing clinical treatment goals [28] and multiple psychological disorders [29,30,31]. Developing intelligent agents that can interact with people and enhance flexibility and realism is possible in virtual environments thanks to AI. Furthermore, these AI agents can now participate in a conversation with patients and even express emotions.

### 3.3. AI in Clinical Diagnostics and Decision-Making

The use of expert systems in the healthcare field is one of the earliest applications of AI, and has also been applied to mental health. An expert system is computer software developed to bring an expert’s expertise and skill into a particular area [32] to assist in the decision-making process [33].

Decision support systems may also be developed to find out and discover patterns and data relationships based on data mining techniques, and hence do not need a prior understanding [34,35]. Many reasoning types are used in decision support systems, such as rule-based reasoning, case-based reasoning, decision tree, and fuzzy systems. In 1970, Stanford University created one of the first therapeutic decision-support systems. The device was developed to recognize bacteria that cause infections and blood-clotting diseases, known as MYCIN [36,37].

MYCIN is a rule-based framework based on typed question-and-answer conversations developed by interviewing experts. While the device worked well in experiments, owing to the computing technology constraints of the day, it was never put to clinical use [36]. Since then, the advances in computing power and AI technologies have significantly increased the capabilities of clinical expert systems. Nowadays, expert systems (using neural network principles and ML techniques) can detect complex patterns and provide interpretations from large amounts of data, which is time- and effort-consuming in the case of manual processing [38,39]. For example, support vector machines [40] is used for the analysis, classification, and recognition of Parkinson’s [41] and Alzheimer’s diseases [42]. Masri and Mat Jani (2012) [43] suggested an AI-based mental health diagnostic expert system (MeHDES) that uses rule-based reasoning techniques to develop knowledge using human expert data about mental health disorders. Fuzzy logic methods have been used to identify the severity of a specific disorder, and fuzzy algorithms have been utilized to identify and develop personalized treatments that consider the patient’s health condition. Via offering a human-like verbal communication interface, AI-enabled augmented reality human avatars with speech recognition and NLP capabilities may also improve expert systems. These programs will provide access to the corpus of specialist information on psychological and medical conditions and be fed with patient medical reports and outcomes of research evidence. Other realistic uses of AI-enabled expert systems involve aid with substance use review, tracking, and contraindication recognition assistance [44].

Authors in [45] describe a framework that allows the development of artificially intelligent agents that can diagnose and overcome medical diagnostic inconsistencies as part of teams regrouping artificial and human medical experts. The advantage of clinical decision support systems based on AI is their ability to handle a high level of complex data, which can help practitioners extract relevant information and make optimal choices. Such programs can also help professionals contend with confusion. Using AI-enabled support systems for clinical decision-making will minimize staff time requirements and help reduce obstacles to restricted specialist expertise in unique fields. Adding to that, as humans are vulnerable to making mistakes due to cognitive failures and stress, in all health care sectors, AI technology can improve skills and decrease human errors in medical decision-making.

### 3.4. Conversational AI

Similar to the chatterbots ELIZA [19] and PARRY [20], conversational AI is the method of allowing computers to converse with us in natural language. Chatbots, voicebots, personal assistants, and other terms have been used to describe them. They can vary slightly from one another. However, one universal attribute that connects them is their capacity to comprehend natural language orders and human demands. These agents would be responsible for carrying out the proposal and engaging in a dialogue on the back end. Conversational AI models may be defined depending on how an agent interprets a natural language (NL) request and maps it to a response.

All the previous works are the start point in conducting our application, but each one has its pros and cons that have to be mentioned. Accordingly, Table 1 summarizes all the previously mentioned works and shows the advantages and disadvantages of each one.

## 4. Mental Health Proposed Diagnosis System for Arabic-Speaking Patients

### 4.1. Purpose and Global Architecture

Our goal was to develop a system that records patient responses to questions during a medical interview (e.g., “I am desperate, and I have no hope in life”). The proposed system provides a detailed psychological diagnosis report of the patient’s condition (e.g., major depressive episode, moderate depressive episode, suicidal, etc.).

**Figure 3:**
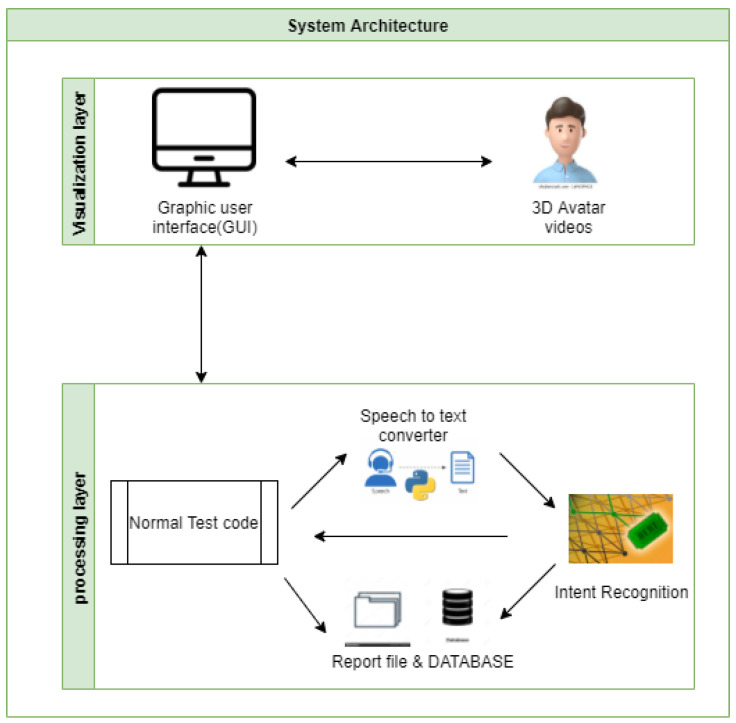
System’s global architecture.

Figure 3 provides a global idea of how the system works. It is divided into two layers: (1) in the visualization layer, the patient interacts with the system via a graphical user interface, and (2) in the processing layer, all his interactions as responses to questions are stored in a database and processed by intent recognition module to generate the final result describing his medical state.

### 4.2. The Input Data and System Patient Interaction

**Figure 4:**
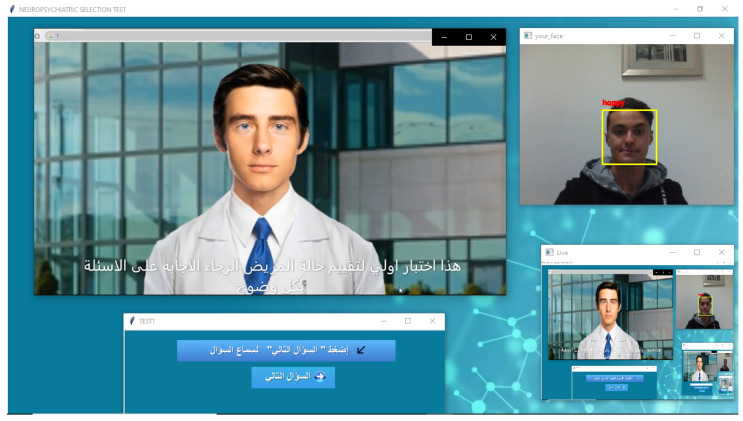
3D human avatar–patient interaction.

To make the system more realistic, we chose to simulate a real-life psychiatric interview where a 3D human avatar, as depicted in Figure 4, plays the doctor and asks the patient the psychiatric questions according to the MINI in its Tunisian Arabic version. The patient, in return, interacts with the avatar by answering the questions vocally.

However, as we mentioned in Section 2.4, the BERT model deals with text and not speech. Thus, to convert the speech to a text, we used a method called speech recognition which refers to automatic recognition of human speech.

**Figure 5:**
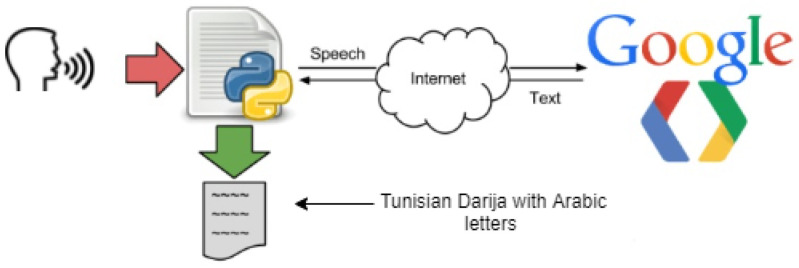
Speech-to-Text process.

Speech recognition is one of the critical tasks in human–computer interaction. Some well-known systems using speech recognition are Alexa and Siri. In our case, we are using the Google Speech-to-Text API with synchronous recognition request, which is the simplest method to perform recognition on speech audio data. It can process up to one minute of speech audio data sent in a synchronous request, and after Speech-to-Text API processes and recognizes all of the audio, it returns the converted text response. It is capable of identifying more than 80 languages to keep up with the global user base, and benchmarks assess its accuracy as 84% [47]. However, in our case, we are dealing with Tunisian “Darija” speech, which is a novelty with Google Speech API, although it offers a Tunisian Arabic option which is in reality different from Tunisia “Darija”, although there are many common words and similarities. The process works by giving the API a speech in Tunisian Darija that it converts and returns back as a text written in Arabic letters, as depicted in Figure 5. Several tests were conducted for the Tunisian dialect (Darija), and 80% of conversion accuracy was achieved in these tests. We had to deal with some limits, such as the complexity of the Tunisian “Darija” (different accents, different languages included in it other than standard Arabic, such as French, Amazigh, Turkish, Maltese, Italian, etc.), which made it very difficult to convert the audio data into a text in a specific language. Our closest option was to convert the speech to text with Arabic letters, although the previously mentioned issues led to some errors such as missing some letters or failing to separate between words due to the tricky pronunciation of the dialect, which we had to take into account while building our dataset. Another limit we had to deal with is blocking the synchronous request, which means that Speech-to-Text must return a response before processing the subsequent request. After testing its performance, we found that it processes audio fast (30 s of audio in 15 s on average). In cases of poor audio quality, our recognition request can take significantly longer, which is a problem we had to deal with when applying the API in our application by reducing noise in the room and enhancing the quality of the microphone.

### 4.3. Dataset

We used the MINI in its Tunisian Arabic version to prepare our dataset. We could not use all of its modules, so we ended up using the five most important ones recommended by the psychiatrists of the military hospital of Tunisia: depression, suicidality, adjustment disorder, panic disorder, and social phobia. We built the dataset by taking the questions of each module and anticipating the answers (e.g., Are you regularly depressed or down, most of the time, nearly every day, over the past two weeks?). This question is depression-related, so we anticipated all of its possible answers (e.g., yes, no, I am depressed, I have been consistently depressed, etc.), and we associated each answer with an intent (e.g., yes—depressed, no—other, I am depressed—-depressed, I have been consistently depressed—depressed, etc.).

This process was challenging in the Tunisian Darija because many answers could have two intents depending on the situation and the nature of the question. For instance, two questions may have the same answer and mean completely two different things. Thus, we risk the repetition of the same answers many times in the same dataset, which may cause overfitting and wrong intent recognition.

Accordingly, to avoid this problem, we used five separate BERT models with a separate dataset for each module instead of one dataset with all the modules. In addition, we ensured that each dataset has unique answers without repetition and with one intent, which justifies the slight imbalance in the dataset between the “intent” class and the “other” class. The “nothing” class includes the misses of the Google Speech-to-Text API, after testing it several times with bad quality audio data (a lot of background noise, bad microphone, etc.), which are added in all the datasets just in case the Speech-to-Text failed under some conditions. It is more of a sign to tell the application user that something wrong in the audio and has to be fixed.

**Figure 6:**
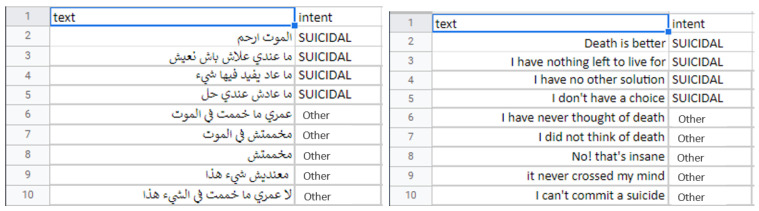
A sample from the suicidality dataset in Tunisian Darija and in English.

Although this justifiable unbalance in datasets may affect the accuracy of the models, especially on the level of the “nothing” class, by providing high-quality audio data and not letting the Google Speech-to-Text API make unwanted errors, we were able to overcome this issue so that the models would not have to deal with the “nothing” class only under very bad conditions, which will be a written warning when the user starts the application. Figure 6 depicts an example of instances in this dataset.

**Figure 7:**
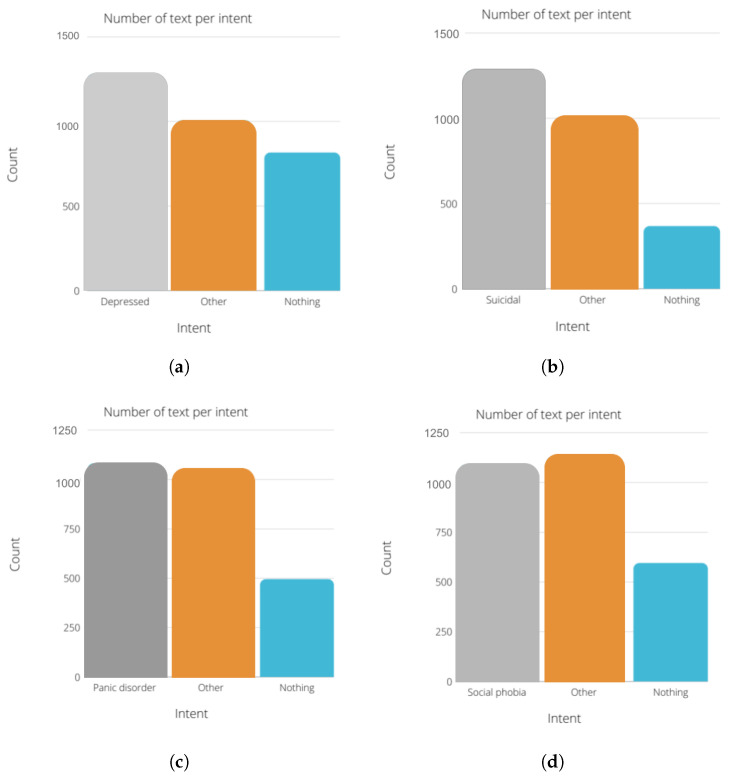

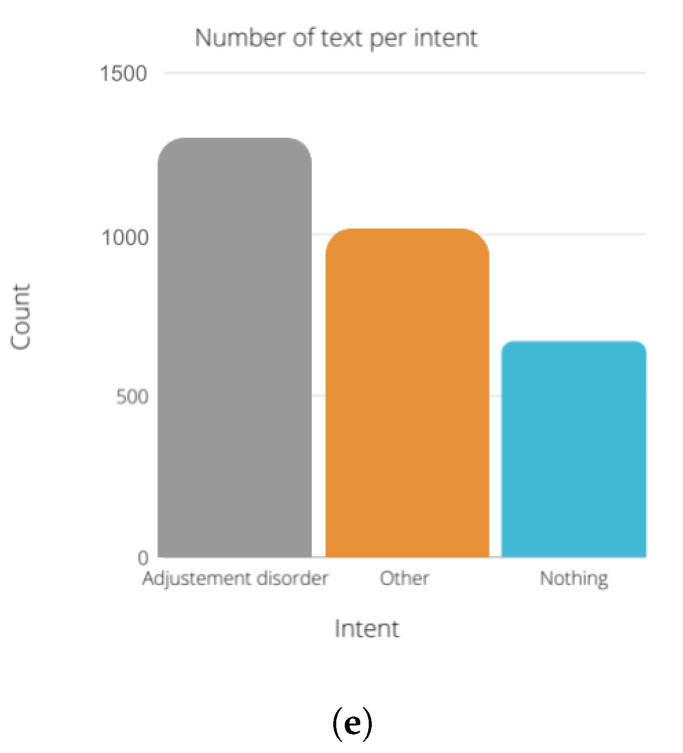
Class distribution of the datasets. (**a**) Depression dataset. (**b**) Suicidality dataset. (**c**) Panic disorder dataset. (**d**) Social phobia dataset. (**e**) Adjustement disorder dataset.

Figure 7 describes the number of text per intent in each module. We chose to use the same amount of “nothing” text for all the modules. The number of instances for the other and the diagnosis state (depressed, suicidal, etc.) are between 1000 and 1500 instances for each module.

#### Data Split

We split our datasets into the training set, validation set, and test set as follows:

- The training set has to include a diverse collection of inputs so that the model can be trained in all settings and predict any unseen data samples.
- Separately from the training set, the validation set is used to make the validation process, which helps us tune the model’s hyperparameters and configurations accordingly and prevent overfitting.

In this study, 80% of the dataset is used for training, while 10% is used for the validation and 10% for testing.

### 4.4. Intent Recognition and Text Classification with BERT

**Figure 8:**
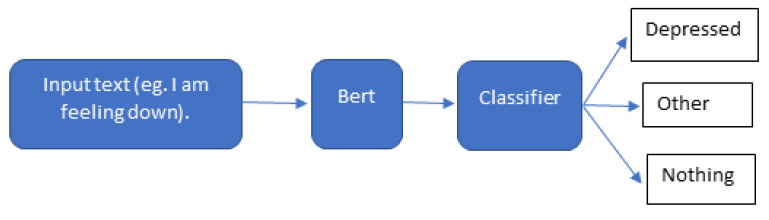
Text classification with BERT.

Our main goal from using the BERT model is to classify the speech of the patient among three classes which are the diagnosis class (depressed, suicidal, etc.), the other class, and the nothing class, as depicted in Figure 8.

**Figure 9:**
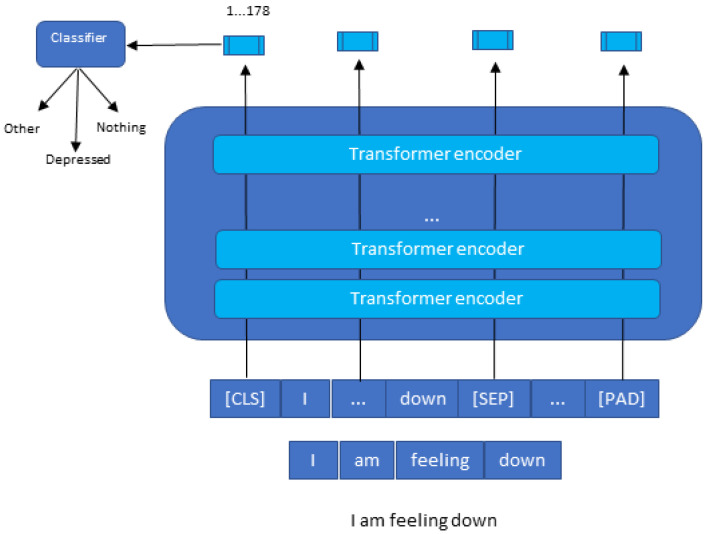
Text classification with BERT in detail.

To understand clearly how the classification with BERT works, Figure 9 explains in detail how the process is carried out.

The BERT model expects a sequence of tokens (words) in the input. In each series of tokens, there are two specific tokens that BERT would expect as an input: CLS, which is the first token of every sequence and stands for classification token, and SEP, which makes BERT recognize which token belongs to which sequence. This token is essential for a next sentence prediction task or question-answering task. If we only have one series, this token will be appended to the end of the sequence.

For example, if we have a text consisting of the following short sentence: “I am feeling down”, first, this sentence has to be transformed into a sequence of tokens (words). As a result, we call this process “tokenization”, as is shown at the bottom of Figure 9. Then, we have to reformat that sequence of tokens by adding CLS and SEP tokens before using them as an input to the BERT model. It is crucial to consider that the maximum size of tokens that the BERT model can take is 512. If they are less, we can use padding to fill the unused token slots with PAD token. If they are longer, then a truncation has to be performed.

Once the input is successful, the BERT model will output an embedding vector of 768 in each of the tokens. These vectors can be used as an input for different NLP applications, such as the classification where we focus our attention on the embedding vector output from the special CLS token. This means the use of the embedding vector of size 768 from CLS token as an input for our classifier, and it will output a vector of size for the number of classes in our classification task. In the Appendix A we provided more code details with python programming language about BERT model creation, training, data splitting, and tokenization.

### 4.5. Evaluation Metrics

To evaluate our system performance and assess the capability of the used models, we considered the metrics namely accuracy, precision, recall, false negative rate (FNR), specificity (which denotes the true negative rate), false-positive rate (FPR), and F1 score (F1) defined as follows:

(1) Accuracy=Numberofcorrectpredictions/Totalnumberofpredictions

(2) Precision=TP/(TP+FP)

TP denotes true positives, and FP denotes false positives.

(3) Recall=TP/(TP+FN)

FN denotes false negatives.

(4) FNR=FN/(TP+FN)

TN denotes true negatives.

(5) Specificity=TN/(TN+FP)

(6) FPR=FP/(TN+FP)=1−Specificity

(7) F1=2×(precision×recall)/(precision+recall)

## 5. Results

### 5.1. Confusion Matrix after Test

**Figure 10:**
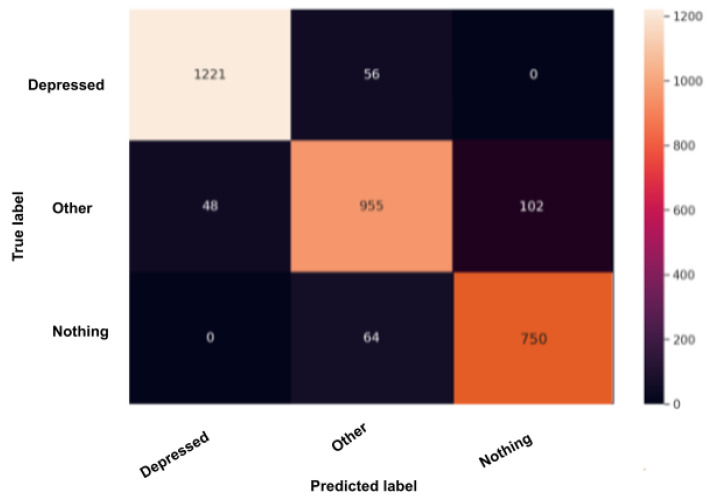
BERT model confusion matrix when using the depression model.

**Figure 11:**
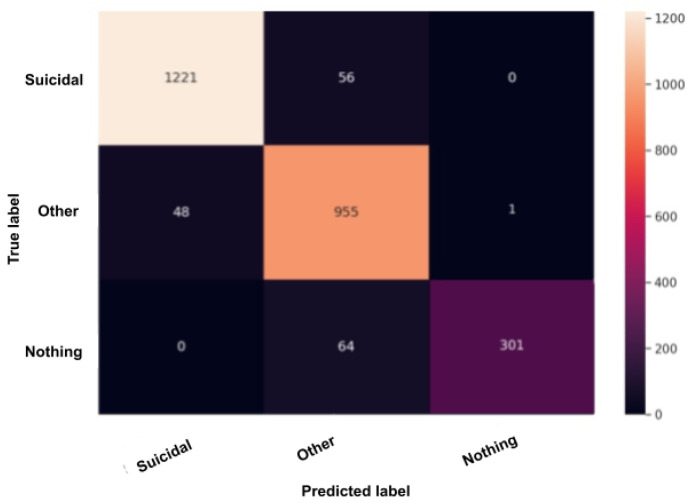
BERT model confusion matrix when using the suicidality model.

**Figure 12:**
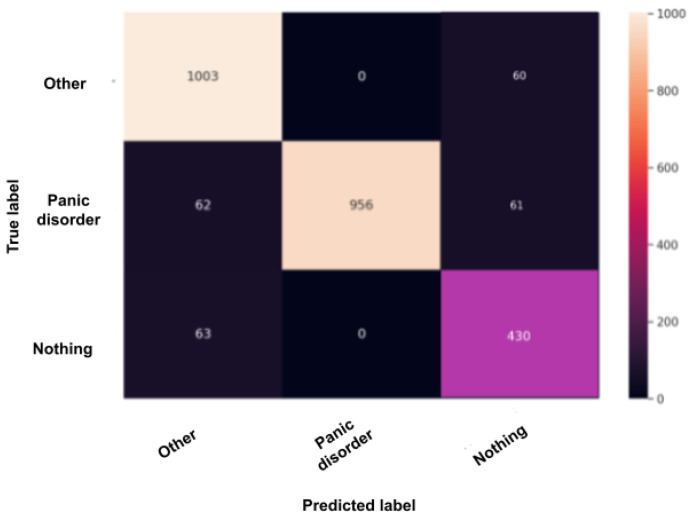
BERT model confusion matrix when the using the panic disorder model.

**Figure 13:**
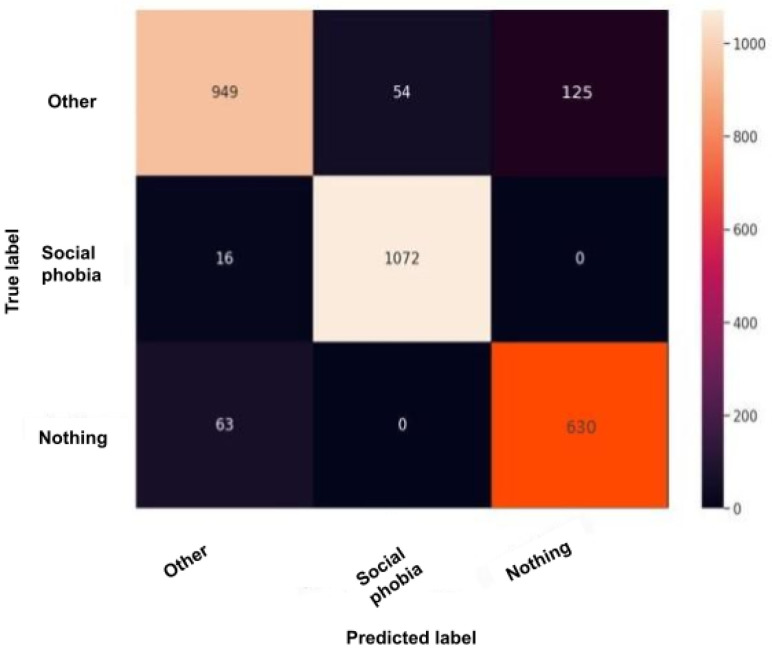
BERT model confusion matrix when using the social phobia model.

**Figure 14:**
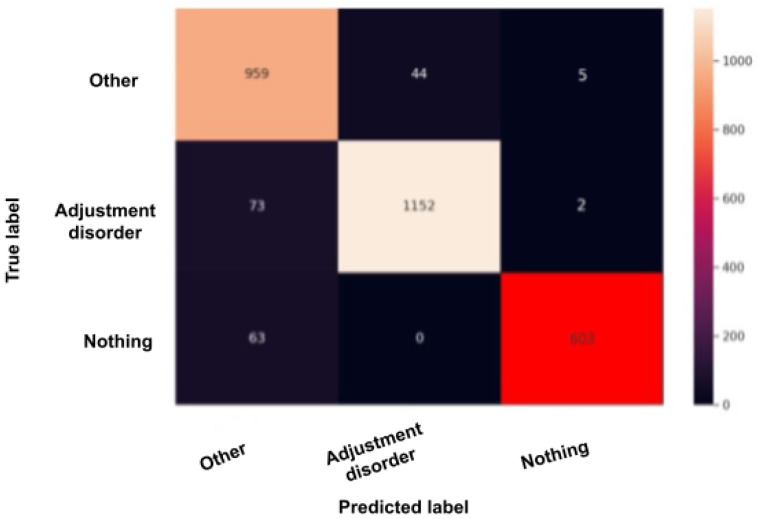
BERT model confusion matrix when using the adjustment disorder model.

The confusion matrix, also called the error matrix, is a table that permits to indicate the performance of an algorithm. Every row of the matrix represents the instances of an actual class, while every column represents the instances in a predicted class or vice versa. If the instances happen to be in the predicted class and the actual class, we have true positives (TP) or true negatives (TN). Otherwise, we have false positives (FP) or false negatives (FN). It makes it easy to confirm whether the system is confusing classes. Figure 10, Figure 11, Figure 12, Figure 13 and Figure 14 are the confusion matrices of each BERT model when undergoing the test set. The lighter the color, the better results obtained. For instance, in Figure 10, the depression model depicts good results, especially with the “depressed” and “other” classes, although there is a higher rate of miss predictions with the “nothing” class. In Figure 11, we obtained the same results as in the depression model (Figure 10) but even better, as the miss predictions (FP and FN) in the “nothing” label are fewer. In Figure 12, when using the panic disorder model, the amount of TP and TN are improving because the colors are becoming lighter, especially with the “other” and the “panic disorder” class, and even the number of miss predictions is decreasing. In addition, in Figure 14, in the adjustment disorder model, the TP is greater than the FP and the FN.

### 5.2. Accuracy after Training and Testing

After training the five models, we had to test their performance using the previously defined metrics. The global results are summarized in Table 2, and detailed results for each model are shown in Table 3, Table 4, Table 5, Table 6 and Table 7. Overall, the accuracy for all models is over 92%, which depicts the high capability of these models to predict all of the classes correctly.

### 5.3. AUC–ROC Curve

**Figure 15:**
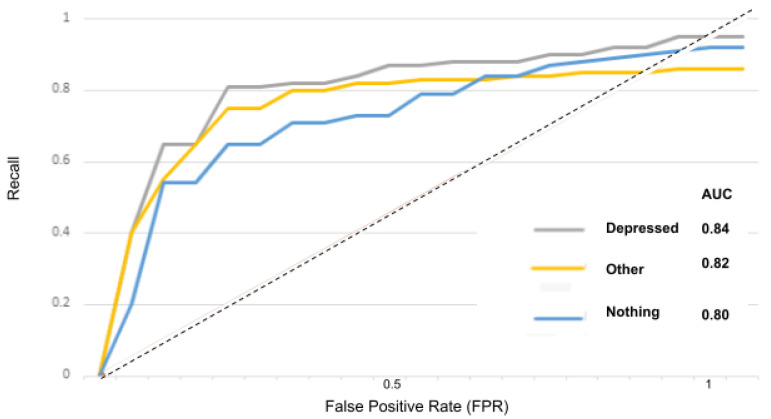
ROC curves when using the depression model.

**Figure 16:**
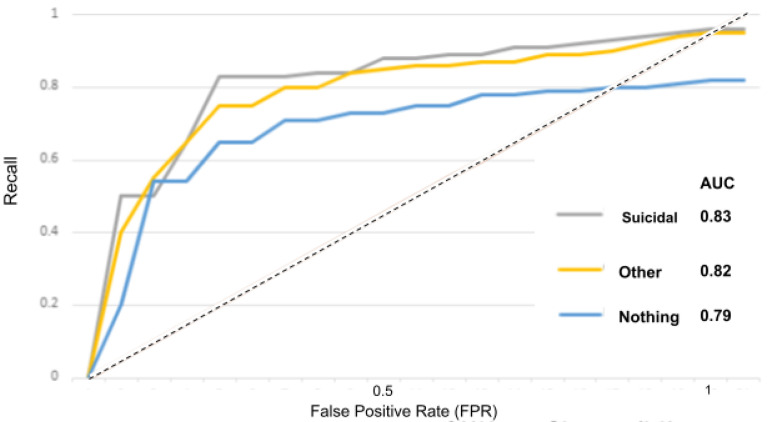
ROC curves when using the suicidality model.

**Figure 17:**
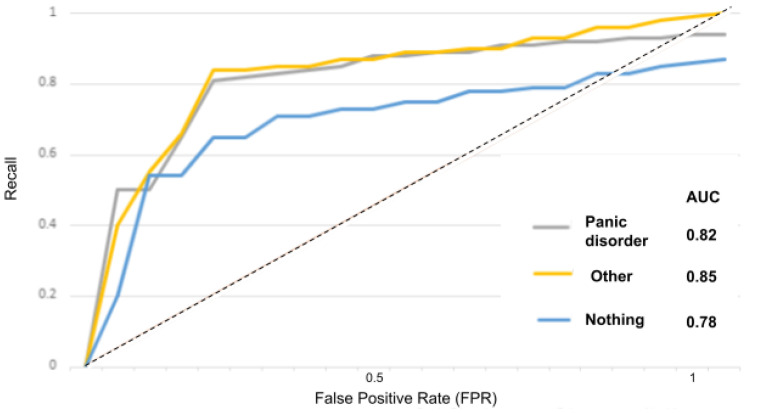
ROC curves when using the panic disorder model.

**Figure 18:**
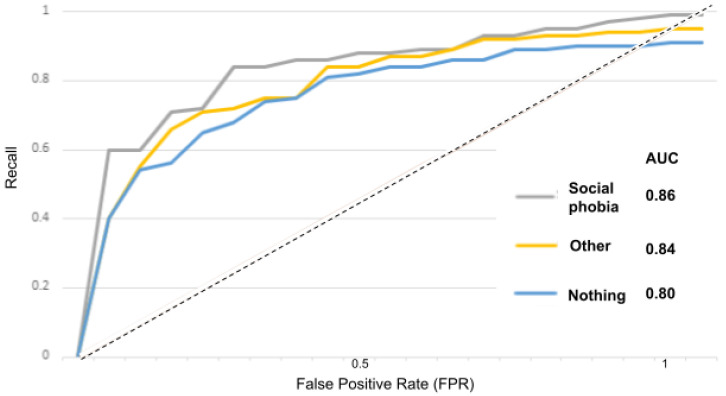
ROC curves when using the social phobia model.

**Figure 19:**
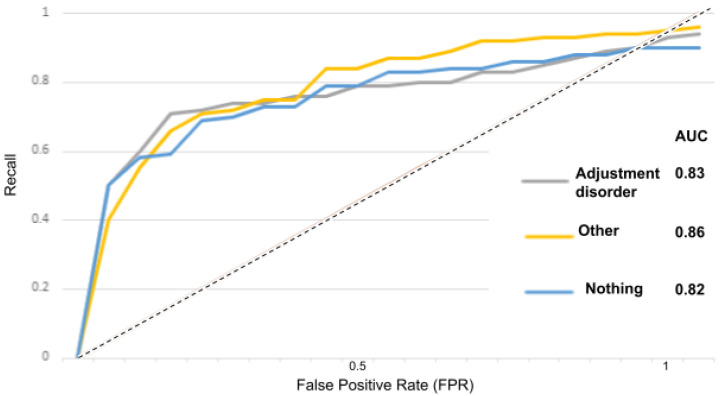
ROC curves when using the adjustment disorder model.

The receiver operator characteristic (ROC) curve is an evaluation metric for models performance. It is a probability curve that plots the recall against the FPR at various threshold values and separates the “signal” from the “noise”, as portrayed in the Figure 15, Figure 16, Figure 17, Figure 18 and Figure 19.

The area under the curve (AUC) measures the ability of a classifier to distinguish between classes and is used as a summary of the ROC curve: the higher the AUC, the better the performance of the model at distinguishing between classes.

In Figure 15 for instance, the AUC score for the “depressed” class is 0.84 and for the “other” and “nothing” classes, the AUC scores are 0.82 and 0.80. With the suicidality model in Figure 16, the AUC score for the “suicidal” class is 0.83, and for the “other” and “nothing” classes we obtained 0.82 and 0.79. With the panic disorder model in Figure 17, the “panic disorder” class is 0.82, and for the “other” and “nothing” classes, we obtained 0.85 and 0.78. With the social phobia model in Figure 18, the “social phobia” class is 0.86, and for the “other” and “nothing” classes we obtained 0.84 and 0.80, and with the adjustment disorder model in Figure 19, the “adjustment disorder” class is 0.83, and for the “other” and “nothing” classes we obtained 0.86 and 0.82.

## 6. Discussion

After finishing the system alongside its graphic user interface, the results from the train and test (accuracy, precision, recall) were very interesting. For instance, train and test accuracy were over 92% for all five tested mental health issues. Accordingly, the next step was to test the system on several patients with real mental health problems to test its reliability, so we first had to guarantee our patient’s approval and their consent to undergo the system, which was granted to us by all means under one condition, which is privacy and without revealing any identities. Afterward, when making the test, the diagnosis results were very satisfying. The patients had already been seeing the doctor for quite some time (we already knew their diagnosis). Although, while interacting with them, they seemed to us as they did not suffer from any problems; even our conversations were ordinary, and we let them interact with the application in private without any intervention. We only used the system to test whether it is precise and gives back a diagnosis similar to the doctors. However, the test on one of the patients occurred as follows: the patient was already diagnosed by the doctor with depression, social phobia, and adjustment disorder, while the system returned that he/she is depressed and has social phobia, ignoring the adjustment disorder part. This preliminary diagnosis may be explained because the patient already knows his/her diagnosis; therefore, the test will not be neutral. Nevertheless, if we were dealing with a new patient who does not know about his/her case or his/her issues, the results would be satisfying even for doctors.

Countless applications have been developed and serve the same purpose as ours does. They can even interact with many different languages, such as English, Spanish, French, and even Arabic; however, to our knowledge, there is no system or application in mental health in the world that can interact with a sixty-year-old Tunisian man with no academic level, little understanding of standard Arabic, and no knowledge of any other language. For that reason, our application makes a difference because it is in the Tunisian dialect, which makes every Tunisian capable of interacting with it. It may need some amelioration, such as offering a treatment or a solution for the diagnosis, because it is essential to help the doctor make an accurate diagnosis and make a clear decision for the treatment. Still, it remains the first of its kind in the country, especially in mental health care, which is very neglected in Tunisia despite the efforts in the field.

## 7. Conclusions

To conclude, the goal of the proposed research is to solve mental health issues using AI. It is a complex problem that has already been approached several times with different techniques, especially ML. This paper focused on building a psychiatric testing system using ML techniques. This system was successfully validated by its highly accurate models; it was tested on actual patients in the Tunisian Military Hospital, and it is intended to be used by the psychiatric department due to its beneficial and promising results that provide a very detailed and accurate patient report. Nevertheless, we can ameliorate this system even further for more results by employing other detection and recognition models such as motion detection, and even heartbeat detection, to obtain a complete and more accurate psychological diagnosis, which can be helpful in decision-making for doctors and patients.

## Figures and Tables

**Table 1:** Summary of the well-known and successful mental health AI technologies.

Technology Name:	Advantages	Disadvantages
ELIZA [19]	Interacts with the patient without bias and lack of judgment.	Was not a smart chatbot by any means, it did not learn or adapt due to its simple script.
PARRY [20]	Models the behavior of a paranoid schizophrenic, has a superior depth of programming and language. The first to pass the Turing Test.	Was not meant to deal with patient problems. It was modeling the behavior of a patient instead.
YOUPER [22]	Helps users recognize monitor, and process their emotions and thoughts. Very affordable.	Only for English-speaking patients.
Woebot [23]	Assists users in self-managing their mental health difficulties.	Speaks only English and limited Italian and its free version is very limited.
Moodkit [24]	Managing depression, anxiety, and stress,	Only for English-speaking patients.
Wysa [25]	Emotionally intelligent chatbot. It helps manage thoughts and emotions. It can diagnose mental health problems with up to 90 percent accuracy.	Only for English-speaking patients.
SimCoach [46]	Very efficient, especially in solving PTSD problems.	Used only in the US military.
Virtual Reality applications	Very proficient and effective.	Very expensive. Was not meant for the public use.

**Table 2:** Accuracy after train and test.

Module	Training Accuracy	Testing Accuracy
Depression	0.9465	0.9281
Suicidality	0.9368	0.9283
Panic disorder	0.9495	0.9400
Social phobia	0.9474	0.9382
Adjustement disorder	0.9299	0.9214

**Table 3:** BERT model with the depression model performance scores.

	Precision	Recall	FNR	Specificity	FPR	F1-Score
Depression	0.96	0.95	0.04	0.97	0.02	0.95
Other	0.88	0.86	0.13	0.94	0.05	0.87
Nothing	0.88	0.92	0.07	0.95	0.04	0.90

**Table 4:** BERT model with the suicidality model performance scores.

	Precision	Recall	FNR	Specificity	FPR	F1-Score
Suicidal	0.96	0.96	0.04	0.03	0.03	0.96
Other	0.89	0.95	0.04	0.07	0.07	0.92
Nothing	0.99	0.82	0.17	0.00	0.00	0.90

**Table 5:** BERT model with the panic disorder model performance scores.

	Precision	Recall	FNR	Specificity	FPR	F1-Score
Panic disorder	1.00	0.94	0.11	1	0.00	0.97
Other	0.89	1.00	0.05	0.91	0.08	0.94
Nothing	0.78	0.87	0.12	0.94	0.05	0.82

**Table 6:** BERT model with the social phobia model performance scores.

	Precision	Recall	FNR	Specificity	FPR	F1-Score
Social phobia	0.95	0.99	0.01	0.96	0.03	0.97
Other	0.92	0.95	0.15	0.95	0.04	0.93
Nothing	0.83	0.91	0.09	0.94	0.05	0.87

**Table 7:** BERT model with the adjustment disorder model performance scores.

	Precision	Recall	FNR	Specificity	FPR	F1-Score
Adjustment disorder	0.96	0.94	0.06	0.97	0.07	0.95
Other	0.88	0.96	0.04	0.92	0.02	0.91
Nothing	0.99	0.90	0.09	0.99	0.00	0.94

**Figure A1:**
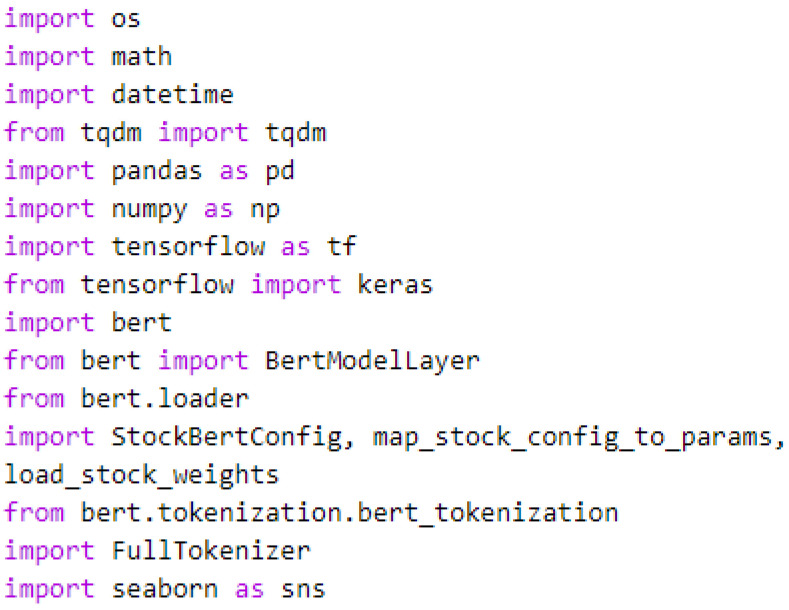
Packages and libraries import.

**Figure A2:**
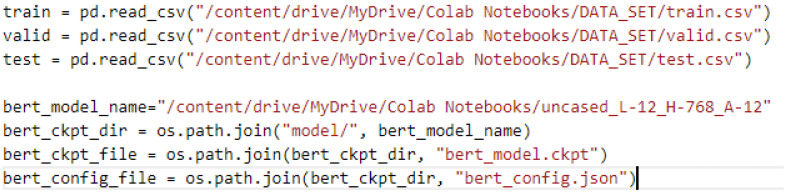
Dataset and model load with Python on Google Colab.

**Figure A3:**
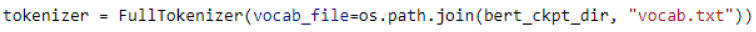
The tokenization code.

**Figure A4:**
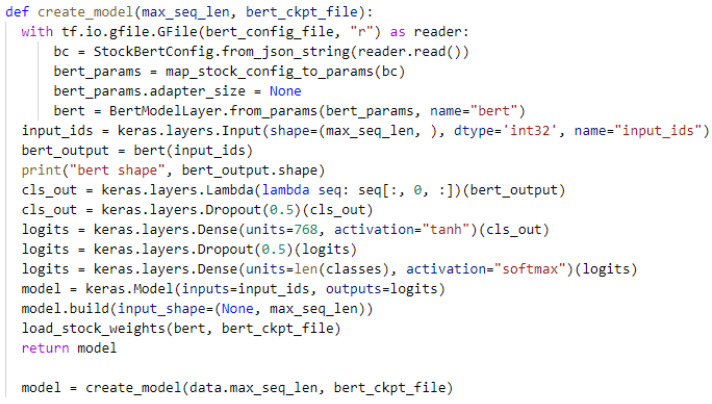
Model creation.

**Figure A5:**
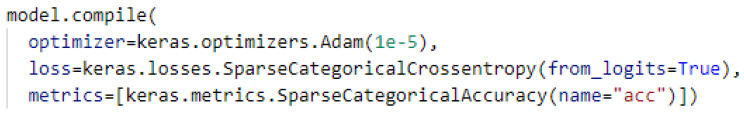
Compiling the model.

**Figure A6:**
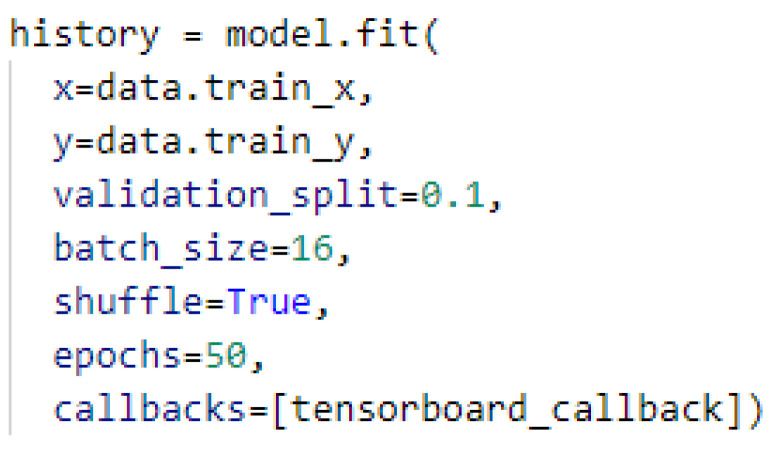
Fitting code.

## Abbreviations

Abbreviations used in this paper are as follows:

NLP	Natural language processing
MINI	MINI Intenational Neuropsychiatric Interview
BERT	Bidirectional encoder representations from transformers
ML	Machine learning
AI	Artificial intelligence
TP	True positives
FP	False positives
TN	True negatives
ROC	Receiver operator characteristic
AUC	Area under the curve

## Appendix A. BERT Model Creation and Fitting

As we are dealing with five different datasets, we had to create also five other models. The following is the method for one of them in Python.

### Appendix A.1. Packages and Libraries

To properly use the BERT model, some packages and libraries had to be imported, such as those depicted in Figure A1.

#### Appendix A.1.1. Dataset and Model Load

Figure A2 depicts the load of the test, train, and validation dataset, and also the model with Python on a Google Colab Notebook.

#### Appendix A.1.2. Tokenization

To utilize a pretrained BERT model, we must first transform the input data into a suitable format so that each phrase can be submitted to the model and the associated embedding can be obtained. Thus, we use the FullTokenizer function as depicted in Figure A3.

### Appendix A.2. Creating the Model

The BERT model had already been created, but we do not have it functioning. We only have its bert-config-file, so we built it according to that bert-config-file, as depicted in Figure A4.

- We started by applying a lambda layer, which is merely another layer that may be invoked directly in TensorFlow. The argument is initially specified within the lambda layer. This value is “seq” in the code excerpt above (lambda seq). In this case, we want a list of all values of seq and that has 0 in it, so we use seq[:,0,:]. Thus, if seq = [1, 2, 3], this lambda layer changes it to [1, 2, 3, 0, 1, 2, 3]. We also specified its shape, which is bert-output, according to the bert-config-file.
- To avoid the overfitting risk, we used the dropout layer with a rate of 50%.
- We used a dense layer with 768 units and the hyperbolic tangent activation function (tanh).
- For the output layer, we opt to use a dense layer with several units equal to the length of the targeted classes and the activation function (softmax).

### Appendix A.3. Compiling the Model

Figure A5 depicts the compilation of the BERT model using different parameters explained as follows:

- The optimizer is an algorithm that adjusts the weights to reduce the loss.
  In our case, we used the Adam algorithm.
- The loss function estimates the discrepancy between the target’s actual value and the predicted value by model. Different issues require different loss functions, and in our case, we used the sparse categorical cross entropy, which is a sort of measure for the distance from one probability distribution to another. The idea is that we want our model to predict the correct class with a probability of 1.0. The more significant the cross-entropy loss, the farther the projected probability is from 1.0. When using cross-entropy, other metrics we might care about (such as accuracy) will tend to improve along with it.
- Accuracy is one of the different measures used to assess a classification problem’s performance. It is calculated by dividing the correct predictions by the total predictions: accuracy = correct predictions/total predictions.
  An accuracy score of 1.0 would be assigned to a model that consistently predicted accurately. All else being equal, it is a reasonable metric to use whenever the classes in the dataset occur with about the same frequency. As a result, we used the sparse categorical accuracy as a metric.

#### Fitting the Model

After we created and compiled the model, we went with the train using our train dataset in Figure A6, using 50 training loops (epochs), hoping that it would be enough.
